# The brain’s response to pleasant touch: an EEG investigation of tactile caressing

**DOI:** 10.3389/fnhum.2014.00893

**Published:** 2014-11-10

**Authors:** Harsimrat Singh, Markus Bauer, Wojtek Chowanski, Yi Sui, Douglas Atkinson, Sharon Baurley, Martin Fry, Joe Evans, Nadia Bianchi-Berthouze

**Affiliations:** ^1^UCL Interaction Centre, University College LondonLondon, UK; ^2^School of Engineering and Design, Brunel UniversityLondon, UK; ^3^Department of Medical Physics and Bioengineering, University College LondonLondon, UK; ^4^School of Psychology, University of NottinghamNottingham, UK; ^5^London College of Fashion, University of the Arts LondonLondon, UK

**Keywords:** affective touch, electroencephalogram (EEG), somatosensation, somatosensory, beta band, tactile

## Abstract

Somatosensation as a proximal sense can have a strong impact on our attitude toward physical objects and other human beings. However, relatively little is known about how hedonic valence of touch is processed at the cortical level. Here we investigated the electrophysiological correlates of affective tactile sensation during caressing of the right forearm with pleasant and unpleasant textile fabrics. We show dissociation between more physically driven differential brain responses to the different fabrics in early somatosensory cortex – the well-known mu-suppression (10–20 Hz) – and a beta-band response (25–30 Hz) in presumably higher-order somatosensory areas in the right hemisphere that correlated well with the subjective valence of tactile caressing. Importantly, when using single trial classification techniques, beta-power significantly distinguished between pleasant and unpleasant stimulation on a single trial basis with high accuracy. Our results therefore suggest a dissociation of the sensory and affective aspects of touch in the somatosensory system and may provide features that may be used for single trial decoding of affective mental states from simple electroencephalographic measurements.

## INTRODUCTION

Several lines of research indicate that touch can have strong influence on our liking of both animated and unanimated objects (Armel and Ramachandran, 2003; Banissy and Ward, 2007; Hamlin et al., 2007; Morrison et al., 2010). It is obvious from everyday life experience but it has also been shown empirically that touch can have a strong impact on social behavior, both in the here and now, but also during the formation of long-term relationships, e.g., the attachment phase during infant development (Harlow and Suomi, 1970; Guest et al., 2009; Essick et al., 2010; Gallace and Spence, 2010; McGlone et al., 2014). Neuroscientific research on somatosensation has traditionally focussed on the neuronal circuits that enable elementary sensory functions of touch (Chapman, 1994; Ruiz et al., 1995; Reed-Geaghan and Maricich, 2011; Wacker et al., 2011; Huggins et al., 2014; McGlone et al., 2014) and their cognitive modulations (Bauer et al., 2006; van Ede et al., 2011) or the affective qualities implicated in pain (Ploner et al., 2002). From studies on pain processing it is generally thought that there is a separation of the ‘analytic’ somatosensory processing stream and the ‘affective’ processing stream (Ploner et al., 2000; Singer et al., 2004). Furthermore, neuroanatomically it has been shown that C-fibers, associated with the subjective experience of pleasant touch (Loken et al., 2009) originating from hairy skin project particularly to structures like the posterior insula and cingulate and prefrontal cortex which have themselves been implicated in processing affective valence in hemodynamic studies (Rolls et al., 2003; McCabe et al., 2008; Bjornsdotter et al., 2009; Gordon et al., 2013). Although electroencephalographic measures have been documented to correlate with valence and hedonism (Saletu et al., 2010; Flo et al., 2011), often on an inter-individual or trait-like fashion, to date no study has investigated the real-time electrophysiological correlates of pleasant touch. Here, we investigate the instantaneous electrophysiological signatures of affective valence in touch with a well-controlled experimental paradigm where subjects were stimulated with a set of different fabrics using a robotic caressing device. This was done on the one hand given the salience of hedonic experiences encountered in daily life when faced with fabrics (e.g., clothes etc.). On the other hand there is growing interest to gather objective, quantifiable data on affective sensations induced by commercial products. (Hughes et al., 2012; Petreca et al., 2013)

The purpose of this study was therefore threefold (a) Can we extract electrophysiological signatures related to the affective qualities of non-nociceptive tactile stimulation? (b) are these affective qualities dissociated from early somatosensory processing? and (c) can the obtained signatures be used as predictive electrophysiological features to decode affective states on a single trial basis?

Whilst neuroimaging techniques with high spatial resolution such as functional magnetic resonance imaging (fMRI) are very useful in uncovering the neuroanatomical circuits underlying the sensation of hedonic valence with tactile stimuli, these are limited by the costs and complexity of combining them with the (tactile) stimulation techniques used here, as well as their low temporal resolution. By contrast, the much lower costs and simpler apparatus necessary to record the electroencephalogram (EEG) could simplify the measurement of direct brain responses in more natural environments. In addition, the high temporal resolution of EEG/MEG allows more specific investigations into the temporal domain and therefore the real-time neuronal processes that determine the sensation of hedonic touch. Thus, an electrophysiological approach to decipher the brain’s response to affective touch is of considerable value to popular areas of consumer economics and may in future studies provide more detailed insights into the neurophysiological interactions underlying the sensation of pleasant touch.

### MATERIALS AND METHODS

### PARTICIPANTS

Thirteen healthy volunteers aged between 21 and 40 years (six males, seven females) were recruited through the departmental online participant recruitment system and provided written consent in line with the university’s ethical guidelines. All participants were right handed and none of them suffered from skin allergies, neurological or psychiatric diseases.

### EXPERIMENTAL SET UP

**FIGURE 1:**
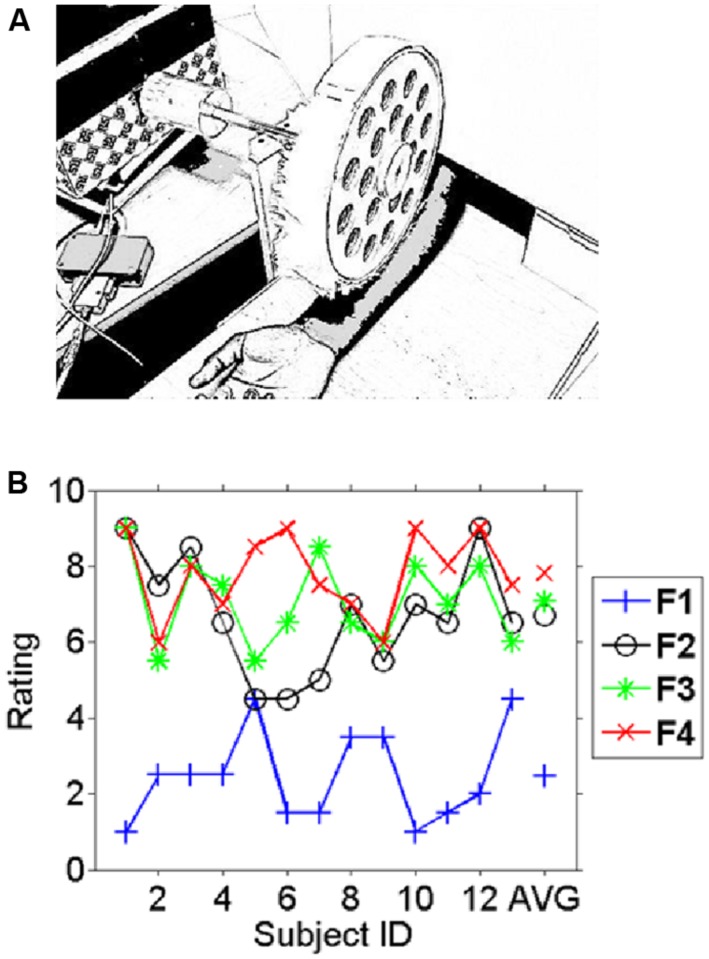
**Experimental setup and behavioral results. (A)** The fabric caressing device (FCD) stimulated the right inner forearm during the experiment by rotational movements. In different blocks four different fabrics were mounted on the drum. Participants had no vision of either fabric or device as this was occluded. **(B)** The average pleasantness ratings (scale 1–9) for the different fabrics across participants (1–13) and the average across subjects on the very right (AVG). A high rating indicates a “pleasant” experience whereas a low rating indicates an unpleasant experience. Subjects consistently rated fabric 1 as the least liked and fabric 4 was on average the most liked fabric. Note the inter-individual variation in the ratings in particular for fabrics 2–4 which can be exploited for correlating pleasantness to the EEG measures.

A motor-controlled fabric caressing device (FCD) presented the fabrics on participants’ right inner forearm. The fabrics were mounted on a wooden drum that was rotated by an electrical motor and could be lowered on the participants’ inner forearm at stimulus onset via a mechanical device that also sent a trigger pulse to the EEG amplifier for precise timing information. At stimulus offset the drum was lifted from the participants’ forearm but kept rotating so as to prevent stimulus-induced brain activity from being potentially confounded with the motor’s electrical field. Visual interferences were minimized by obscuring the vision of the participants with a cardboard wall. A cut-out window enabled participants to insert their arm as shown in **Figure 1A**. The temperature in the experimental room was maintained at 18–20°C. The speed of caressing was kept between 2 and 4 cm/s which was kept uniform throughout the experiment. The circular shape of the FCD implied that with equally spaced four quadrants for the four fabrics, the contact area for stimulation was uniform (∼4–5 cm^2^). The weight of the rotating wheel was counterbalanced with an adjustable weight on the shaft, so that the indenting force on the forearm was uniform with optimum distance between the subject’s forearm and rotating wheel, such that the fabric just touched the forearm. This setup had been tested in a pilot study that is presented in supplemental material.

### MATERIAL

The selection of the fabrics used for the EEG study was based on a behavioral pilot study of 18 subjects (six Males, 12 Females, aged between 18 and 44 years, see supplemental material for details of experiment and results). This group of participants was different from the one used for the EEG study. In the behavioral study, a set of 10 fabrics was used, five of which were different types of ‘fur’ with different hair length (called here after the H-set) and five had a more net-like structure of different degrees of granularity (called hereafter the N-set). This set of fabrics provided different levels of softness and roughness. We describe the methods and results of this pilot study in detail in the supplemental material.

A set of four fabrics reflecting different levels of likeability were selected based on the findings of the pilot study. For the fabrics used in this EEG study, from the most liked to the most disliked, the selected fabrics were: a long haired synthetic fur (Fabric 4), a short haired synthetic fur (Fabric 3), a heavy nylon crinoline (Fabric 2), and a loosely woven lamé and wool textile (Fabric 1). We selected these fabrics to (a) obtain a good range of valence ratings, (b) have some fabrics that are clearly rated as either pleasant or unpleasant (to enhance contrast), and (c) have some fabrics that are rated heterogeneously amongst participants.

### TASK

Strips of the four selected fabrics were mounted on the device and presented in a random blocked order for 2 s with a 2 s rest period in between trials. Each fabric was presented in two consecutive blocks of 25 trials each, separated by 5 min breaks. Subjects were instructed to rate the four selected fabrics on a 9-degree scale to indicate their degree of likeability (1 most disliked and 9 most liked). The rating was performed both at the beginning and at the end of the recording session (where 1 was low valence and 9 was high valence).

### RECORDINGS

Electroencephalogram data were acquired using a 32-channel MR-compatible Brain Products amplifier, BrainAmp at 1000 Hz with standard settings. An active electrode setup (BrainAmp ActiCap) with the extended 10–20 system was used. One electrode was placed underneath the right eye to enable calculation of the vertical EOG.

## DATA ANALYSIS

The behavioral ratings of each of the 13 subjects were analyzed using a repeated measurement analysis of variance (ANOVA) and paired *t*-tests for specific contrasts.

All electrophysiological data analyses were performed using the FieldTrip toolbox (Oostenveld et al., 2011). Data were epoched from -1 to 2 s around stimulus onset. Artifact removal was performed in two stages. First, trials with excessive artifacts (electronic artifacts or infrequent movements causing very large noise) were removed from the dataset after visual inspection (using ‘rejectvisual’). Next, a principal component analysis (PCA) was run over the remaining data (whole epochs) and oculomotor as well as other non-physiological artifacts were removed. Finally, residual artifacts were removed (by eliminating the respective trial) using rejectvisual again. All this was done over the entire session irrespective of experimental condition.

### TIME FREQUENCY ANALYSIS

A time-dependent frequency analysis using a Hanning window with a window length of 0.4 s was performed for frequencies from 2 to 48 Hz in steps of 2 Hz. To assess the effect of *stimulation per se*, the mean and variance in each time-frequency-channel ‘bin’ were calculated and a *t*-test (within subject and across conditions) was computed as a comparison between peri-stimulus time-frequency bins and a baseline period from -0.4 to -0.2 s (see, e.g., Bauer et al., 2006). The resulting *t*-values were then averaged across subjects to reflect a statistical measure of event-related synchronization and event-related desynchronization (ERS/ERD; see, e.g., Pfurtscheller et al., 1997). For all analyses on the electrophysiological correlates of tactile sensation reported here, we first smoothed the resulting power-spectra (and *t*-spectra) with a boxcar function over three consecutive bins in the temporal domain (0.3 s) and three bins (6 Hz) in the frequency domain to account for inter-individual variations in spectral peaks (van Pelt et al., 2012).

### INFERENTIAL STATISTICS

Firstly, we focused our analysis on an a priori region of interest, the stimulus-induced alpha-/beta-suppression (often also referred to as mu-suppression) which is the most prominent neuronal correlate of tactile stimulation (Bauer et al., 2006; van Ede et al., 2011). Secondly, for a more general approach to extract electrophysiological correlates of affective tactile sensation without a priori hypotheses and to therefore account for the multiple comparison problem we chose a cluster-randomisation approach (Maris and Oostenveld, 2007) that effectively corrects for parallel tests (multiple comparison problem) in space (electrodes), time and frequency (thousands of time-frequency-channel combinations). This test currently does not support the calculation of ANOVA’s and therefore we proceeded in the following way to extract statistically significant effects of experimental condition and subjective pleasantness.

Given the interindividual variation of subjective pleasantness ratings across the fabric conditions (see **Figure 1B**), the statistical analysis for testing for (1) the effects of the experimental manipulation of fabric conditions and (2) the subjective pleasantness of touch followed two different paths.

For the first, we selected the (on average) most and least preferred fabrics as a first approach to analyze the data in terms of the difference of induced brain responses by experimentally manipulated condition (by paired *t*-test). This has two advantages: (1) these were the only conditions that were consistently rated as more or less preferred by all participants in the sample, (2) choosing the most extreme conditions should maximize statistical power. Hence, with this test we extracted those electrophysiological signatures that significantly differed with respect to those experimental manipulations (identical for all participants) that yielded the largest subjective difference (on average). This was implemented by a mass-univariate paired *t*-test for those two conditions and (for the whole brain analysis) subsequent permutation tests with multiple comparison correction for type I error on the cluster-level (Maris and Oostenveld, 2007).

**FIGURE 2:**
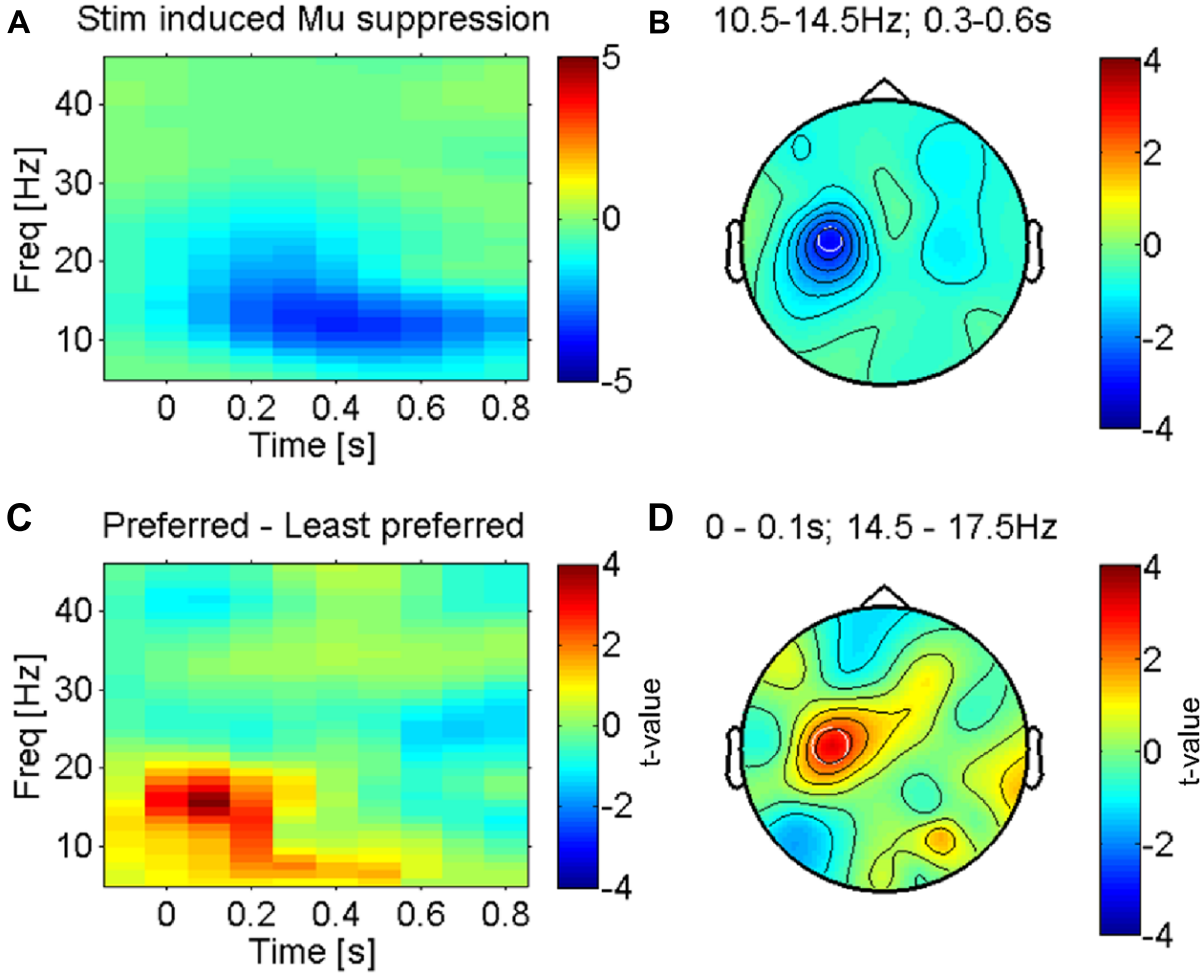
**Somatosensory mu-suppression in response to tactile caressing of the forearm. (A)** Time-frequency representation of stimulus induced suppression of somatosensory mu-oscillations, the most prominent brain response to afferent stimulation (from electrode ‘C3’ as indicated in **B**). Averaged across all conditions (fabrics). **(B)** Topography of this effect (10.5–14.5 Hz and 0.3–0.6 s after stimulus onset) against baseline, averaged across conditions. The effect is dominant over the left somatosensory cortex (electrode ‘C3’), contralateral to the side of stimulation (right forearm), as expected. **(C)** Time-frequency profile of the direct statistical comparison (dependent sample *t*-test) between oscillatory power in ‘C3’ for preferred and least preferred fabrics. A significant difference emerges just after stimulus onset in the higher mu-band (14.5–17.5 Hz). **(D)** Topography of this effect is consistent with the well-known mu-suppression (peak in ‘C3’) and therefore presumably the somatosensory cortex, see text.

For the second, in order to directly test for the effect of subjective pleasantness (irrespective of the specific fabric condition) and to take the data of all four conditions into account, we additionally performed for individual subjects a regression analysis of induced brain responses on their pleasantness ratings (that showed considerable interindividual variation; see **Figure 2**). To this end, we first conducted a mass-univariate regression analysis (within subject) with the pleasantness ratings as the predictor variable on each time-frequency-channel data point (separately, and separate regression analyses for each subject). The regression slopes of this analysis (frequently referred to as first-level analysis in SPM type analyses) were then subjected to a one sample *t*-test (*t*-test for dependent samples contrasting an empirical dataset against a null-distribution, testing effectively against 0) with correction for multiple comparisons on the cluster level.

Whilst these two tests were conducted independently from each other (i.e., the results of one not informing the other), in practice the tests would not be statistically independent. This is because (as can be seen in **Figure 2**) the pleasantness ratings are (not unexpectedly) correlated across participants (the correlation between conditions ranked by average pleasantness ratings in the sample and individual ratings was 0.58, i.e., ∼34% shared variance). Whilst there is a considerable amount of intersubject variation, the response to the most and least pleasant fabrics is relatively common in the sample. Hence, in order to check whether the so extracted electrophysiological features might largely be driven by, e.g., a bimodal distribution of the electrophysiological features and ratings (non-homoscedasticity), we additionally show scatterplots with the associated correlations of the electrophysiological features over pleasantness ratings across participants. Here, we subtracted the individual means of each subject (across conditions) for both ratings and power values – to eliminate non-condition-specific inter-individual differences, and to exploit the differential expression of the electrophysiological and likeability measures for all four different fabrics.

### SINGLE TRIAL ANALYSIS

We next aimed to assess whether these features that reliably distinguished between pleasant and unpleasant stimulation on the population level could be used also to predict the different fabric conditions on a “real time” or trial to trial basis. A Bayesian logistic classifier approach was employed for this purpose (van Gerven et al., 2009a,b). There is a twofold advantage to this method. Firstly it uses prior spatio-temporal information from the data while computing the prediction model (van Gerven and Simanova, 2010; van Gerven et al., 2010). Secondly, it can be easily used for more than two classes. The input to this algorithm was a set of beta or mu-power features highlighted during the time-frequency analysis. This feature set was computed as power spectral density values in the beta band (26–30 Hz). Similarly a feature set for mu-rhythm (10.5–14.5 Hz) was computed. The Donders Machine Learning toolbox (DMLT) as integrated within the Fieldtrip toolbox was used for computing the predictive models for the single trial analysis. The computed feature set was used to train the classifier on the trial data in two scenarios: (a) for the least and most liked fabrics (b) for all the four fabrics. In scenario (a), the output of the classifier was compared with the true labels for each trial belonging to the least liked or the most liked category to calculate the prediction accuracy. In scenario (b), the output of the classifier was compared with the fabric type. The accuracy, expressed as a percentage is a measure of the ability of beta features to correctly predict the condition to which each trial belongs. Fivefold cross-validation procedure was conducted for evaluation of the prediction accuracy values. The average ‘accuracy of prediction’ over the cross-validation folds was used as the metric to evaluate the robustness of the beta features for predicting the single trial’s fabric category.

## RESULTS

### BEHAVIORAL RESULTS

The fabrics were chosen based on an unpublished pilot study that is presented in the supplemental material. Subjects rated the fabrics in this study individually before and after the EEG experiment. **Figure 1B** shows the individual average ratings for all fabrics used. Intra-class correlation as a measure of reliability for the two ratings was carried out using SPSS v.20 (ICC, average measure, two-way mixed model). The results show that the participants were highly consistent over the two ratings of the same fabric [ICC = 0.945, 95% confidence interval (CI): 0.904–0.968, *p* < 0.017]. The likeability ratings differed significantly between conditions (*F* = 45.2, *p* < 10^-11^). While Fabric 1 was clearly and consistently the least liked, Fabric 4 was fairly consistently the most liked fabric. Generally differences in ratings between the more pleasant fabrics (Fabrics 2, 3, and 4) were small in comparison to their difference with the least-liked (Fabric 1). Simple (paired *t*-) tests showed for the comparison of Fabrics 1 and 2 (the least and second least liked) a highly significant difference (t = -6.8, *p* < 10^-5^), whereas Fabrics 3 and 4 (the second most and the most liked fabrics) were not significantly different (*t* = -1.0, *p* > 0.1) and ratings for Fabrics 2 and 4 were just significantly different (*t* = -2.3, *p* < 0.05). We therefore restricted the main comparison for the categorical differences in electrophysiological responses to the least and the most liked fabric as those would yield the most interpretable contrast (difference in rating: *t* = -9.8, *p* < 10^-6^), but see the correlation analysis below for a different strategy.

### MU-SUPPRESSION OVER SENSORIMOTOR CORTEX

The most prominent feature of tactile stimulation was a suppression of low-frequency alpha-/beta- (also termed mu-) oscillations in electrodes lying over the left somatosensory cortex (see **Figures 2A,B** for the average across all fabric conditions), contralateral to the side that was stimulated (right forearm). This effect is well known and reflects the presumably most robust effect of tactile stimulation in somatosensory cortex, covering several sensorimotor areas but possibly with a dominant source in the primary somatosensory cortex (Crone et al., 1998; Cheyne et al., 2003; Bauer et al., 2006; Feurra et al., 2011).

We investigated the modulation of this effect by the least and most preferred fabrics (see methods). **Figure 2C** shows the time courses of power over electrode C3, an electrode that is known to be located above the left (primary) sensorimotor cortex (Pfurtscheller et al., 1997). Significant differences between the most and least preferred fabrics (red and blue respectively for Fabrics 4 and 1 in **Figure 1B**) emerge just around the time of stimulus onset (see **Figure 2D** for the direct statistical comparison) and reveal a larger suppression of mu-oscillations at a peak-frequency for the least preferred Fabric 1. The early onset of this difference suggests that the effect is largely driven by the onset of contact and hence likely reflects an afferent response property (see, e.g., Garrido et al., 2007). The topography of the maximum difference (see **Figure 2D**) strongly suggests that this effect originates in the sensorimotor cortex, likely the primary somatosensory cortex. This effect was, however, not correlated with participants’ subjective pleasantness ratings across all four conditions (*r* = 0.25, *p* > 0.1, uncorrected).

### ELECTROPHYSIOLOGICAL CORRELATES OF PLEASANTNESS

**FIGURE 3:**
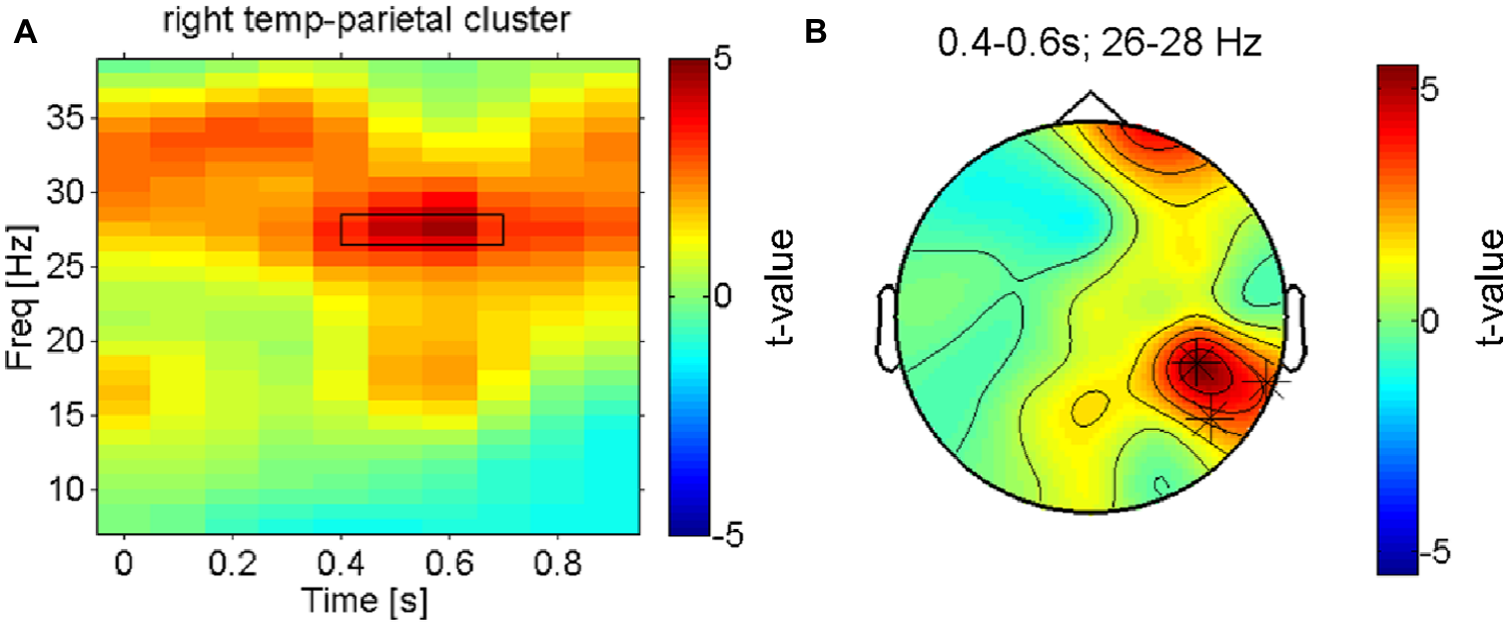
**Right hemisphere beta-oscillations distinguish most pleasant from least pleasant sensations. (A)** Time-frequency-representation of the contrast between the most and least preferred fabric conditions shown for the significant cluster in right temporo-parietal electrodes. The rectangle indicates the time-frequency window that the cluster-analysis showed as significant. **(B)** Topographical result for the statistical comparison of preferred vs. least preferred fabrics in the significant time-frequency window (26–28 Hz, 0.35–0.65 s as marked in **B**). The asterisks indicate the electrodes that form the significant cluster that survived correction for multiple comparisons. Besides these right parietal electrodes, a right frontal electrode is also strongly modulated (n.s. when corrected).

After the assessment of the mu-suppression (the dominant electrophysiological signature of early somatosensory processing), we carried out a more complete search to identify the neuronal processes underlying pleasant vs. unpleasant tactile stimulation – without any a priori constraints and accounting for the multiple comparison problem – using a cluster-randomisation approach (Maris and Oostenveld, 2007). To maximize statistical power given the considerable inter-individual variation in pleasantness ratings across the four conditions, we first contrasted the electrophysiological responses to the (on average) most vs. least pleasant fabric for all participants using a dependent samples *t*-test. The only cluster that was found to be significantly different (at *p* < 0.05, corrected) was a right lateralized beta-band effect (∼25–30 Hz; see **Figure 3A**) in an electrode cluster over the right temporo-parietal cortex (see **Figure 3B**). The topography of the (unthresholded) test-statistic reveals that this effect was localized in the right temporo-parietal electrode cluster and had a companion over the right frontal cortex (a single electrode) that had a highly similar time-frequency profile of the contrast but did not reach the multiple-comparison corrected significance threshold.

**FIGURE 4:**
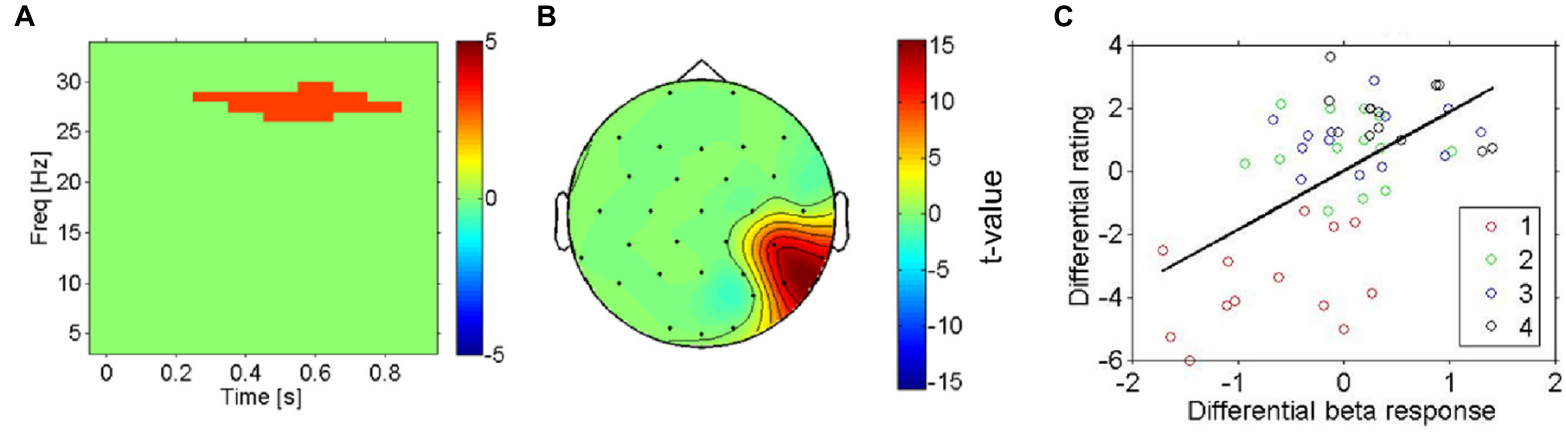
**Right hemisphere beta-oscillations as correlates of likeability (all four conditions). (A)** Result for regression analysis using mass-univariate regression analysis with the pleasantness ratings as the predictor variable on each time-frequency-channel data point. The thresholded *t*-statistic (adjusted for multiple comparisons) reports the significance of the regression analysis for all time-frequency bins. The highlighted time-frequency window survived the multiple comparisons. **(B)** Topographic representation (thresholded) of the *t*-statistic for the regression analysis in the significant cluster **(A)**. **(C)** Scatterplot of induced beta-oscillations in right temporo-parietal electrodes (see **A**) against likeability ratings of each fabric (in different colors). The mean across conditions was subtracted from each subject (both EEG and ratings) to reveal differences between conditions rather than inter-individual differences of their averages across conditions. There is a strong positive correlation between the beta-response and the likeability ratings, indicating that the effect shown in **(A,B)** does not reflect a mere physical difference in stimulation.

Next, we aimed to search more directly for electrophysiological correlates of subjective pleasantness including all four conditions, taking the intersubject variation of pleasantness ratings into account. The statistical significance of regression slopes of the individual induced spectral responses (for each time-frequency-channel bin, see methods) on each participant’s pleasantness ratings were tested with a one-sample *t*-test against 0. This analysis shows a highly similar pattern of results to the previous test (most vs. least liked) in that it replicates the presence of a beta band effect (**Figure 4A**, thresholded) on temporo- parietal electrodes (see **Figure 4B**, thresholded). Higher beta-power was associated with more pleasant stimulations – and this was true not only for the categorical difference between the most and least pleasant fabrics, but also for the correlation of beta-power with the subjective ratings across all four conditions (*r* = 0.58, *p* < 0.01, uncorrected, for the temporo-parietal cluster), showing that this is a more continuous effect (see **Figure 4C**).

### SINGLE TRIAL PREDICTION

**FIGURE 5:**
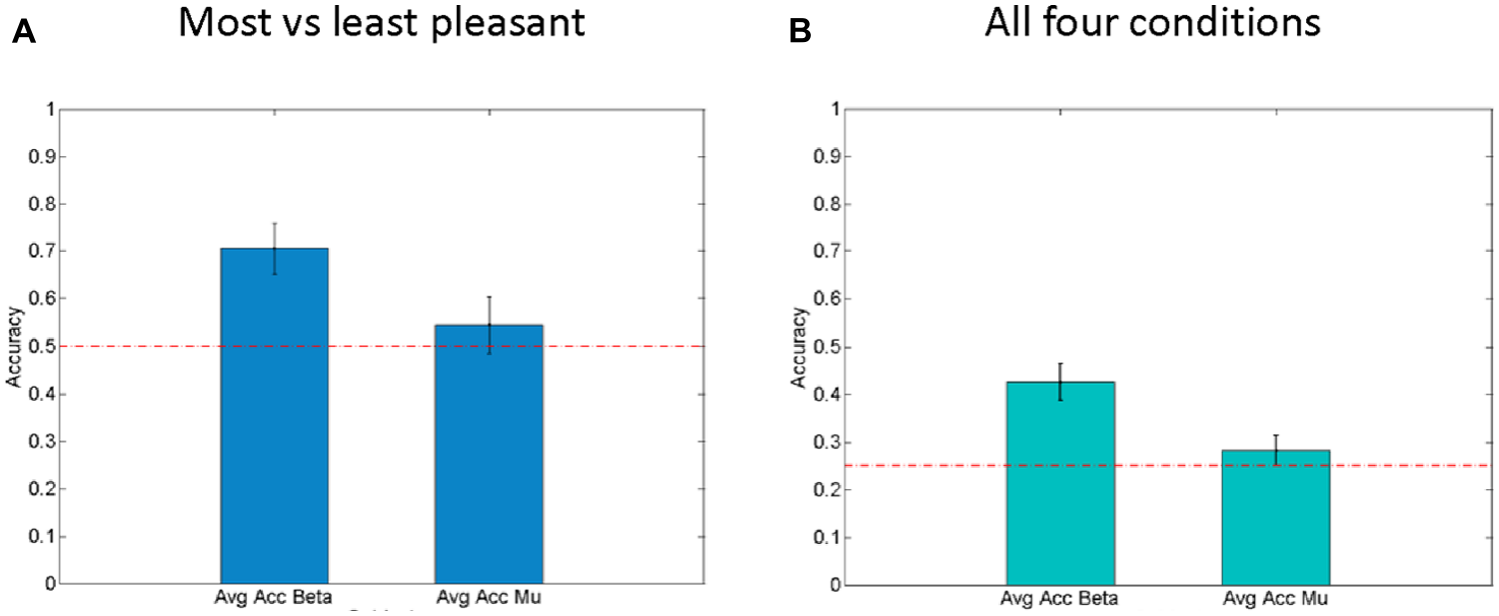
**Right hemisphere beta-oscillations as correlates of affective valence. (A)** Average results for fivefold cross validation for Single Trial level predictions of “Least liked vs. Most liked fabric” using both beta-modulation (left) and mu-suppression features. Standard error of the mean is shown as error bars. Chance level (0.5) is indicated by the horizontal line. **(B)** Average results for fivefold cross validation for Single Trial level predictions of all four conditions using both beta modulation (left) and mu-suppression features. Standard error of the mean is shown as error bars. Chance level (0.25) is indicated as horizontal lines.

In order to evaluate the effectiveness of these beta-band-oscillations (and mu-oscillations for control) as a feature for decoding the affective response of a participant on a single trial basis, a Bayesian logistic classifier was used. This classifier was trained to either classify individual trials as (a) pleasant (most liked) vs. unpleasant (disliked) responses to a fabric, or to classify (b) trials from all four conditions (fabrics) using a one vs. the others framework (Schlögl et al., 2005; Bashashati et al., 2007; Lotte et al., 2007). A fivefold cross validation procedure was used to test the performance of the classifiers and the average accuracy is shown in **Figure 5**. For the first case (a), the average accuracy of prediction ranges was 70.6% across all subjects (see **Figure 5A**) with SEM of 3.2%, at a chance level of 50%. This indicates the robustness of beta power features to discriminate between individual pleasant and unpleasant stimulation trials. By contrast, for the classification based on mu-power over early somatosensory cortex, the performance was considerably worse, namely 54.5% with a SEM of 2.9%.

In the second case (b), using all four fabrics (see **Figure 5B**), with chance level being 25%, the average prediction accuracy across the entire population is 42.5% with SEM of 2.7%. Interestingly, for the mu suppression, the accuracy of prediction for the four fabric conditions is relatively low with an average of 28.2% and SEM of 1.4% (see **Figure 5B**). Concerning the classification of all four conditions, it needs to be said that the separation of subjective pleasantness for these four conditions is considerably weaker, hence it should be of no surprise that in this second case (b), performance drops considerably. Nevertheless, the single-trial results from the Bayesian logistic classifier analysis support the results obtained from classical inferential statistics in that they show that particularly the right parietal beta-band oscillations can be used to discriminate states that are associated with pleasant vs. unpleasant stimulation (by fabrics) and that they do so more than the mu-suppression over contralateral somatosensory cortex.

## DISCUSSION

In this paper we assessed the electrophysiological correlates of hedonic valence in tactile perception. Our results show that right hemispheric beta-band oscillations measured over parietal electrodes allow to differentiate between pleasant and unpleasant tactile sensations and that this does not merely reflect the effect of a different physical stimulation: Whereas early somatosensory mu-oscillations were also significantly modulated by stimulation with different fabrics – these were not (significantly) correlated with subjective likeness ratings and this effect is therefore more likely to reflect differences in low-level physical features rather than affective states. This is also confirmed by the single trial analysis in which a Bayesian logistic classification analysis based on mu-oscillations over contralateral somatosensory cortex shows relatively low performance, indicating inadequacy of the mu-features to accurately predict the affective states induced by caressing with different fabrics. By contrast, the right hemisphere sustained beta-oscillations were tightly correlated with subjective perception and are therefore considered to reflect the representation of hedonic valence during caressing. This is in line with the fact that the Bayesian Logistic classifier reached relatively high accuracy in the classification of pleasant vs. unpleasant trials based on the beta-feature.

Previous electrophysiological studies investigated the affective component of touch using pain scenarios (Ploner et al., 2000, 2002). While early somatosensory cortex has been shown to be implicated in pain processing (Ploner et al., 2000; Hauck et al., 2007), several studies have shown different aspects of the involvement of lower and higher somatosensory, or polymodal, areas (Ploner et al., 2002; Singer et al., 2004). Two recent studies that manipulated the emotional state of participants (Senkowski et al., 2011; Yoshino et al., 2012) showed that beta-suppression over the sensorimotor cortex was enhanced for increased levels of pain even for physically identical nociceptive stimuli. While we also found enhanced mu-suppression over the sensorimotor/somatosensory cortex for stimulation with one of the less pleasant fabrics, this effect was not significantly correlated to subjects’ subjective pleasantness ratings. This could reflect a dissociation in the processing of painful vs. non-nociceptive unpleasant tactile stimuli with respect to the potential involvement of early somatosensory cortex in representing qualia like affective valence.

We did find, however, an increase in right temporo-parietal and frontal electrodes particularly in the beta-range for stimulation with the most pleasant fabric relative to the least pleasant fabric and this was strongly correlated with participants’ self-reports of pleasantness across all four experimental conditions. Since the profile of subjective ratings for the four fabrics showed considerable variation across subjects (and therefore this correlation is not trivial) this latter effect seems to be tightly correlated to participants’ hedonic valence experience. Indeed, previous electrophysiological studies have also found that temporo-parietal beta-activity is correlated with emotional states in different tasks (Ray and Cole, 1985; Schutter et al., 2001).

It is difficult to infer the source of these enhanced beta-oscillations measured over temporo-parietal and frontal electrodes, however the localized spatial peaks of these effects (in a cluster of contiguous electrodes for the temporo-parietal effect) may suggest a more localized origin (Nunez and Westdorp, 1994) rather than a spatially non-specific widespread effect. Previous fMRI studies (Rolls et al., 2003; Bjornsdotter et al., 2009; Voos et al., 2012; Gordon et al., 2013) have revealed posterior insula, medial frontal and cingulate cortex as brain structures involved in hedonic experiences of tactile stimulation (see also for neuroanatomic evidence; Loken et al., 2009) and the topography of this beta-activity seems consistent with sources from these locations. Furthermore, inherently hedonic tactile stimuli have been shown to activate inferior prefrontal regions. In particular afferent projections of somatosensory cortex onto the orbitofrontal cortex (OFC), inferior frontal gyrus (IFG), and adjacent anterior frontal operculum (Hagen et al., 2002) have been associated with this sensation. While we also measure frontal beta-activity that changes with subjective states of pleasantness, this effect did not reach statistical significance when adjusting for multiple comparisons; hence, although there may be suggestive evidence of observing such structures in our data, our conservative analysis approach and the inverse problem preclude strong inferences concerning the involvement of these brain regions for our results.

Concerning more practical implications, the decoding of affective states from individuals is non-trivial. In different contexts, researchers have shown the difficulty of this endeavor (Hertenstein et al., 2009; Kleinsmith and Bianchi-Berthouze, 2013). The enhanced beta-oscillations not only significantly covary with the individual participant’s subjective rating of pleasantness for the utilized fabrics when averaged across trials, but they also allow us to reliably classify fabric conditions associated with most pleasant vs. least pleasant stimulation on a single trial basis. The analysis for the four conditions as included here faces the challenge that, in most subjects, the overlap of the subjective pleasantness (likeability) across all four conditions was considerable, therefore imposing severe constraints on the chance to separate these fabric conditions accurately (our analysis only allowed categorical classification according to fabric condition). The performance of these classification results also has to be seen in the context of these relatively simple recordings, without an electrically shielded chamber and with a relatively low-density EEG setup (32 channels). Hence, these results are in the first line a *proof of principle* and provide a very promising perspective for the development of an affective brain computer interfaces (aBCI), where hedonic responses to fabric touch are predicted from a neural response (Frantzidis et al., 2010; Hurst et al., 2012; Kim et al., 2013). In previous research, visual and auditory stimuli (facial recognition, movie clips, or music fragments) have been successfully used to decode the emotional state (Balconi and Lucchiari, 2006; Yuan-Pin et al., 2010; Dan et al., 2011; Yisi and Sourina, 2012), as well as nociceptive stimuli for pain (Schulz et al., 2011). A limitation of the current approach is also that we did not ask participants to rate the pleasantness of these stimuli on individual trials, but only at the beginning and at the end.

With respect to the location of the caressing, the forearm was selected based on previous studies. C-mechanoreceptive units, also called CT (C-tactile) afferents found on the hairy, non-glabrous skin have been found to have closer relations to limbic functions than to sensorimotor functions (Vallbo et al., 1999; Olausson et al., 2010). CT afferents are quite slow as compared to Aβ afferents (faster and are more associated with discriminative or sensorial response of touch). Neuroimaging studies show activation of the posterior insular as well as somatosensory areas S1 and S2 when both CT and Aβ afferents are stimulated (Olausson et al., 2010). Behaviorally, affective responses to tactile stimulation were more prevalent when either the calf, forearm, thigh, i.e., hairy areas (rich of CT-afferents) were stimulated (Essick et al., 2010). Hence, the FCD was designed to deliver the tactile stimuli to the inner forearm to produce the strongest affective response.

One question that might arise concerning the hedonic sensation during caressing is that of memory processes that may contribute to such sensations, e.g., through classical conditioning. Since we do not know the individual history of the participants used here, we cannot make qualified statements as to whether such mnemonic processes may impact on the affective responses to stimulation with different fabrics.

Taken together our results suggest that scalp electroencephalographic measurements can reveal subjective hedonic valence in the form of right parietal (and possibly frontal) beta-oscillations. These signals appear to be related to the representation of affective valence for tactile stimuli whereas neural activity in presumably early somatosensory cortex seems to reflect the more mechanical aspects of tactile processing. The specific role of beta-oscillations in processing of emotional information remains to be clarified, one possibility being that these reflect the enhanced network-activity (Gross et al., 2004; Donner et al., 2007) of higher order somatosensory areas such as, e.g., the posterior insula, cingulate and prefrontal cortex that have been suggested to encode hedonic experience in touch (Rolls et al., 2003; Bjornsdotter et al., 2009; Gordon et al., 2013).

Importantly, even in this relatively simple experimental setup, beta-oscillations classified individual trials with pleasant stimulation from trials involving unpleasant stimulation with good accuracy, suggesting their usefulness to more directly measure affective states in a wide range of studies from neuromarketing (Plassmann et al., 2012; Solnais et al., 2013) to the research of affective behavior in social and economic tasks (Sanfey et al., 2006; Fliessbach et al., 2007; Glimcher et al., 2008).

## Conflict of Interest Statement

The authors declare that the research was conducted in the absence of any commercial or financial relationships that could be construed as a potential conflict of interest.
