# Supplementary material for: The brain’s response to pleasant touch: an EEG investigation of tactile caressing
Source: Front Hum Neurosci. 2014 Nov 10;8:893. doi: 10.3389/fnhum.2014.00893 (PMC4226147; doi:10.3389/fnhum.2014.00893)
Supplement: Supplementary file 1 [file Data_Sheet_1.DOCX]

***Supplementary Material***

**The brain’s resonance to pleasant touch: an EEG investigation of tactile caressing**

**Harsimrat Singh*^acd^, Markus Bauer*^b^, Wojtek Chowanski^a^, Yi Suyi^a^, Douglas Atkinson**^e^**, Sharon Baurley^c^, Martin Fry^d^, Joe Evans^d^, Nadia Bianchi-Berthouze^a**^**

^a^UCLIC, University College London,WC1E6BT, UK

^b^ School of Psychology, University of Nottingham, University Park, Nottingham, UK

^c^School of Engineering & Design, Brunel University, London, UK

^d^Department of Medical Physics and Bioengineering, University College London, London, UK

^e^London College of Fashion: University of the Arts London, London, UK

*** These authors contributed equally**

****Correspondence**: Nadia Berthouze, UCLIC, University College London, WC1E6BT, UK;

Email: [n.berthouze@ucl.ac.uk](mailto:n.berthouze@ucl.ac.uk), Phone: +44 20 7679 0690

1. **Behavioral Study**

The selection of the fabrics used for the EEG study was based on a behavioral study in which 18 subjects (6 Males, 12 Females, aged between 18-44 years) participated. This group of participants was different from the one used for the EEG study. In the behavioral study, a set of 10 fabrics was used, 5 of which were different types of ‘fur’ with different hair length (called here after the H-set) and 5 had a more net-like structure of different degrees of granularity (called hereafter the N-set). This set of fabrics provided different levels of softness.

The experimental setup was exactly similar to the EEG study. Each fabric was presented to the subject’s forearm in a random sequence using a fabric caressing device (FCD). Subjects were asked to self-report the tactile experience in the form of ratings on a 10-degree scale in ascending order of pleasantness. The trial was repeated three times with a 5 minute break between each trial to account for habituation.

**1.1 Results of the behavioural study:**

Figure A shows the average ratings per participants per fabric over the three trials. Intra-class correlation as a measure of reliability for the three trial ratings was carried out using SPSS v.20 (ICC, average measure). The results show that the participants were highly consistent over the three ratings of the same fabric (ICC=0.861, 95% confidence interval [CI]: 0.821–0.893, p<.001). The scores over the three trials were hence added up obtaining a total appreciation score per fabric and per participant.

A one-way repeated-measures ANOVA was used to identify differences between fabrics in their level of pleasantness. The test was performed on the total appreciation scores from the three trials. As expected, the results showed an effect of fabric (F (9, 153) =33.995, p<.01, η_p_^2^ = .667). Post-Hoc t-tests with Bonferroni correction were conducted (see table 1). The results showed that fabric H4 obtained significantly higher ratings than any other fabric but fabrics H2 and N5. Fabrics N1 and N2 obtained significantly lower ratings than all the other fabrics but fabric N3. All the others fabrics had scores in the middle range. The two intermediate fabrics H5 and N4 shared a similar pattern of appreciation even if H5 seems to have received slightly higher scores (Figure B). Their ratings were both only significantly lower than H4 and higher only than the ones for N1, N2 and N3.

Following this analysis, H4, N4, H5, N1 were selected for the EEG experiments to have a good distribution of ratings, a representation of the two types of fabrics and a low inter-individual differences in the fabric scores (See figure A and B). 3N was not included in the selection because it was quite rigid and thus it showed to be impractical to mount on the caressing device due the circular shape of the caressing wheel.


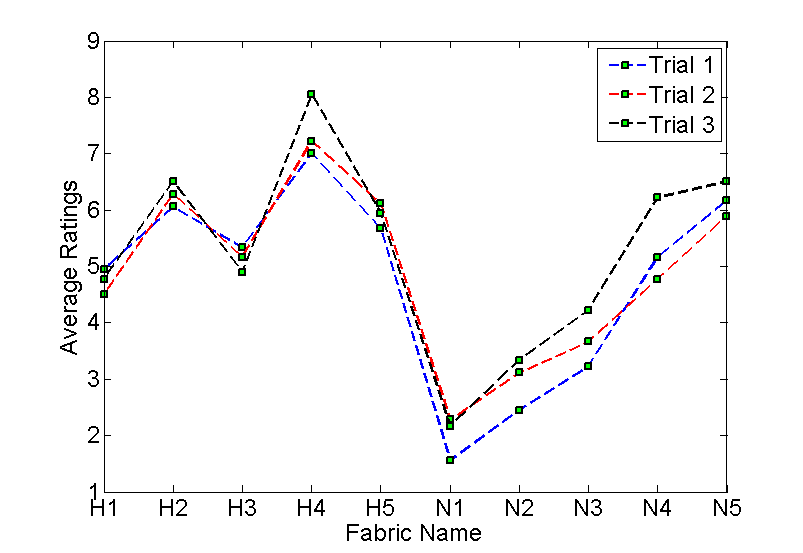


Figure A


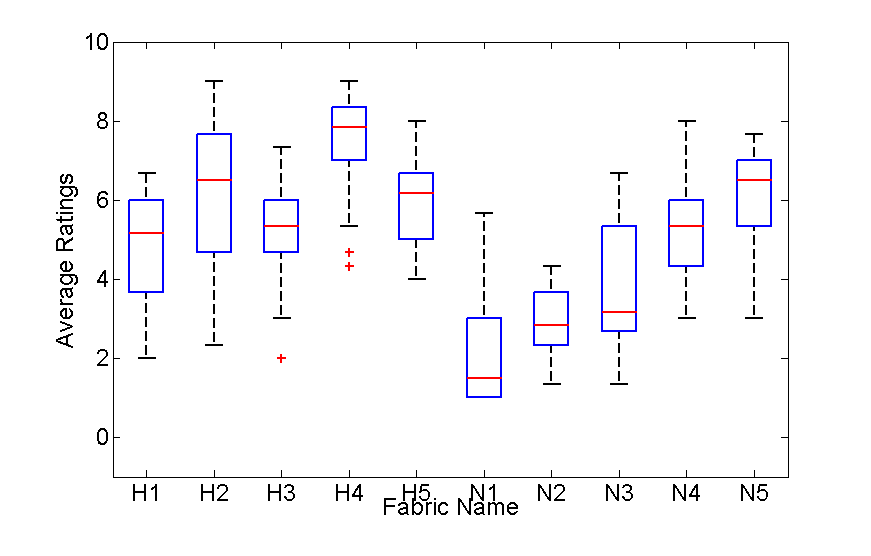


Figure: B

Table 1

|  | H1 | H2 | H3 | H4 | H5 | N1 | N2 | N3 | N4 | N5 |
| --- | --- | --- | --- | --- | --- | --- | --- | --- | --- | --- |
| H1 |  |  |  | - |  | + | + |  |  |  |
| H2 |  |  |  |  |  | + | + | + |  |  |
| H3 |  |  |  | - |  | + | + |  |  |  |
| H4 | + |  | + |  | + | + | + | + | + |  |
| H5 |  |  |  | - |  | + | + | + |  |  |
| N1 | - | - | - | - | - |  |  |  | - | - |
| N2 | - | - | - | - | - |  |  |  | - | - |
| N3 |  | - |  | - | - |  |  |  | - | - |
| N4 |  |  |  | - |  | + | + | + |  |  |
| N5 |  |  |  |  |  | + | + | + |  |  |

**Legends:**

Figure A: the average rating profile for each fabric for the population (n = 18) for each of the three trials.

Figure B shows the boxplots of average ratings from 3 trials for each of the ten fabrics for all 18 subjects. Whilst Fabric H4 was consistently rated higher than others, the ratings of N1 were lowest across the population. The circled fabrics are selected for the EEG study.

Table1: The table shows the results of the Post Hoc tests. A '+' symbol (in a red cell) indicates that the fabric on the row has received significantly higher scores than the fabric in the column. A '-' symbol (in a blue cell) indicates the fabric on the row has received significantly lower scores than the fabric in the column.
